# The Effects of Serping1 siRNA in α-Synuclein Regulation in MPTP-Induced Parkinson’s Disease

**DOI:** 10.3390/biomedicines11071952

**Published:** 2023-07-10

**Authors:** Min Hyung Seo, Sujung Yeo

**Affiliations:** 1 Department of Meridian and Acupoint, College of Korean Medicine, Sang Ji University, Wonju 26339, Republic of Korea; cstcl@naver.com; 2 Research Institute of Korean Medicine, Sang Ji University, #83 Sangjidae-Gil, Wonju 26339, Republic of Korea

**Keywords:** serping1, α-synuclein, MPTP, Parkinson’s disease, colon

## Abstract

Our understanding of the gastrointestinal system in the pathophysiology of Parkinson’s disease (PD) has grown considerably over the last two decades. Patients with PD experience notable gastrointestinal symptoms, including constipation. In this study, the effects of knocked-down serping1, associated with the contraction and relaxation of smooth muscle and inflammation responses, by applying the serping1 siRNA were investigated in 1-methyl 4-phenyl 1,2,3,6-tetrahydropyridine-induced PD mice in an α-syn change aspect. In the result, serping1 expression was knocked down by the treatment of serping1 siRNA, and decreased serping1 induced the decrease α-syn in the colon. Furthermore, the changes in α-syn aggregation were also examined in the brain, and alleviated α-syn aggregation was also observed in an *serping1* siRNA treatment group. The results indicated that serping1 siRNA could ease synucleinopathy related to the gastrointestinal system in PD. This study also raises the possibility that serping1 siRNA could alleviate α-syn aggregation in striatum and substantia nigra regions of the brain.

## 1. Introduction

Parkinson’s disease (PD) is a progressive neurodegenerative disorder that primarily affects the motor system. It is characterized by symptoms such as tremors, muscle rigidity, and impaired balance [1,2]. However, PD is also characterized by common nonmotor symptoms, including cognitive and psychiatric symptoms, autonomic dysfunction, and swallowing difficulties [3,4,5]. PD is characterized by the accumulation of abnormal protein aggregates, particularly α-synuclein (α-syn), in specific regions of the brain, including the substantia nigra (SN) and striatum (ST) [6,7,8]. The presence of increased α-syn in the SN and ST is closely associated with the neurodegenerative process underlying PD [9,10]. The aggregation of α-syn disrupts normal cellular functions, triggers inflammation, and induces oxidative stress, leading to the progressive degeneration of neurons in these regions [11,12,13]. The prevalence of PD is increasing globally, emphasizing the need for a deeper understanding of its underlying mechanisms and potential treatment options.

Understanding of the role of the gastrointestinal system in the pathophysiology and the possible cause of PD has grown considerably over the last two decades. Constipation is a common symptom in PD, and is a notable gastrointestinal symptom, developing long before motor symptoms appear [14,15,16]. Patients with PD also complain of gastrointestinal symptoms, including dysphagia, early satiety, nausea, and bloating [17,18]. In this study, a neurotoxin for a PD model, 1-methyl 4-phenyl 1,2,3,6-tetrahydropyridine (MPTP), generally used for evaluating the dysmotility in the stomach, small intestine, and colon, was used [18,19].

In the other characteristics of PD, abnormal α-syn aggregation in Lewy bodies underlies PD pathology, and α-syn in colon tissue is demonstrated prior to the onset of PD [20,21]. This suggests that the gastrointestinal system may be involved in the early stages of PD pathogenesis. The distribution of synucleinopathy is related to gastrointestinal symptoms along the entire gastrointestinal tract, and α-syn might infiltrate the central nervous system as soluble α-syn oligomers [16,22]. The immunohistochemical staining methods showing the Lewy-type α-synucleinopathy may distinguish PD colon from the control reliably [21,23], and α-syn pathology has also been observed prior to the development of motor symptoms in colonic tissues [20].

Meanwhile, serping1, called C1 inhibitor (C1INH), controlling complement C1, is a serine proteinase inhibitor family G1, and regulates the kallikrein–kinin system and plasminogen activation contributing to inflammation [24]. The upregulations of Serping1 are observed in an active tuberculosis, HIV-1+, and the monocytes treated with IFN-α,β and γ [25,26]. In the kinin system, bradykinin receptor 1 activates endothelial nitric oxide synthase and induces local edema formation and fluid extravasation [27,28]. Based on the reports, serping1 could be associated with the contraction and relaxation of smooth muscle and inflammation response. Previous studies have also reported that serping1 is increased in PD and is associated with α-syn in the brain [29]. Neuroinflammatory disorders include Alzheimer’s disease, PD, and multiple sclerosis [30,31].

In this study, serping1 is knocked down by administering serping1 siRNA in the colon, and how this affects α-syn expression change in PD is investigated, based on the expression pattern of serping1 associated with PD. And founded on the possibility that α-syn, as soluble α-syn oligomers, might infiltrate the central nervous system, α-syn oligomer expression changes in brain were also investigated. It is anticipated that this study can contribute to alleviating synucleinopathy and abnormal α-syn aggregation in PD.

## 2. Materials and Methods

### 2.1. PD Induced by MPTP and Making Groups

Eleven-week-old C57BL/6J male mice (25–27 g; DBL, Daejeon, Korea) were used in this study. The groups were divided into four groups (*n* = 6/group): control group (CTL); MPTP-treated and only lipofectamine-treated group (NC); MPTP-treated and lipofectamine and *Serping1* siRNA-treated group (SER); and *N*-acetylcysteine-treated group (NAC). To induce PD, MPTP-HCL (20 mg/kg of free base; Sigma, Burlington, MA, USA) was injected intraperitoneally three times every 2 h in the NC, SER, and NAC groups. On the other hand, phosphate-buffered saline (PBS) instead of MPTP-HCL was injected three times at an interval of 2 h in the CTL group. And then, materials for testing were injected intraperitoneally 2 h after the third injection (materials at 4th time: CTL group, PBS treatment; NC group, only lipofectamine treatment; SER group, lipofectamine and serping1 siRNA (5 mg/kg) treatment; NAC group, NAC (150 mg/kg) treatment). The sacrifice of mice anesthetized using Alfaxan was performed 7 days from the treated day. Transcardial perfusion was also conducted with cold PBS for Western blotting. All animal experiments in this study were approved by Institutional Animal Care and Use Committee (IACUC) of Sang Ji University (IACUC protocol approval #2021-8).

### 2.2. Preparation of the Materials

In the SER group, 1 mM *Serping1* siRNA (5′-CAC CUA UGU GAA UGC AUC U-3′) (Bioneer, Daejeon, Republic of Korea) and lipofectamine reagent (Invitrogen, Waltham, MA, USA) were diluted in PBS with diethylpyrocarbonate (DEPC). *Serping1* siRNA (5 mg/kg; mouse weight) and lipofectamine (2.3%) were injected per mouse. In the NC group, only lipofectamine reagent (2.3%) was diluted in PBS with DEPC in the same manner of the SER1 group and was injected. In the CTL group, only PBS with DEPC was injected. In the NAC group, NAC (150 mg/kg; Sigma Aldrich, Burlington, MA, USA) in PBS with DEPC was injected. As NAC is known to decrease cellular damage by free radicals [32], NAC treatment was performed to build a positive control for comparison with the SER group.

### 2.3. Immunohistochemistry

The brains and colons of mice were resected and fixed in 4% paraformaldehyde for overnight at 4 °C. The brains and colons were dehydrated with sucrose for 24 h at 4 °C. Cryosectioned brains and colons were cut using a cryomicrotome in the coronal direction and in the transverse direction in each (40 μm thickness). Sectioned tissues were washed with cold PBS and incubated in 3% H_2_O_2_ in PBS (pH 7.4) for 15 min. Then, they were incubated with bovine serum albumin (3%) and Triton X-100 (0.3%) in PBS for an hour prior to incubation with anti-α-syn antibody (1:500; Novus Biologicals, Littleton, CO, USA) or anti-Serping1 (1:2000; Cloud clone Corp., Houston, TX, USA) as the primary antibody. The sections were treated with a biotinylated anti-rabbit IgG and an avidin–biotin-peroxidase complex using an ABC kit (Vector Laboratories, Newark, CA, USA). Stained Serping1 and α-syn were developed using diaminobenzidine (0.003% 3,3-diaminobenzidine) hydrogen peroxide (0.03% in 0.05 M-Tris) solution (pH 7.0).

### 2.4. Western Blotting

The bilateral ST and SN regions were extracted from the brains and colonic regions from the intestines of mice perfused with cold PBS, and the tissues were homogenized in radioimmunoprecipitation assay buffer (20 mM) for 30 min on ice. After being centrifuged at 4 °C for 20 min, the supernatant of the samples was quantified by bicinchoninic acid assay and separated by electrophoresis. Separated proteins were transferred to polyvinylidene difluoride membranes (Pall Corp., Port Washington, NY, USA), and the membranes were blocked with 3% bovine serum albumin (GenDEPOT, Katy, TX, USA). Then, the membranes were incubated with primary antibodies: anti-Serping1 (1:2000; Cloud clone Corp., Houston, TX, USA), α-syn antibody (1:500; Novus Biologicals, USA), and anti-β-actin (1:5000; Santa Cruz Biotechnology, Dallas, TX, USA) antibodies. Thereafter, the membranes were washed with Tris-buffered saline (pH 7.4) containing 0.1% Tween-20 (TBST) and incubated with the appropriate secondary antibodies: anti-mouse (1:5000; Abcam, Cambridge, UK) or anti-rabbit (1:5000; Abcam, Cambridge, UK) IgG antibodies. The antigen–antibody complexes were visualized using Alliance Q9 Micro (UVITEC; Cambridge, UK) equipment for chemiluminescence imaging.

### 2.5. Immunofluorescence

Colonic regions obtained from cryosectioned colon were washed with cold PBS and incubated in 0.3% Triton X-100 for 30 min. After incubation in blocking buffer (1% bovine serum albumin, 5% goat serum in PBS) for an hour, colonic sections were incubated with the primary antibodies; mouse anti-α-syn (1:500; BD Biosciences, Franklin Lakes, NJ, USA) and rabbit anti-Serping1 (1:2000; Cloud clone Corp., Houston, TX, USA). Then, secondary antibodies, goat anti-mouse IgG(H+L) fluorescein isothiocyanate (FITC)-conjugated (CUSABIO, Houston, TX, USA), and goat anti-rabbit IgG (H+L) tetramethylrhodamine (TRITC)-conjugated (Novex, St. Louis, MO, USA) were used. Colonic sections were treated with 4′,6-diamidino-2-phenylindole (DAPI; 1 μg/mL). Photographic documentation was conducted using a Nikon X-cite series 120Q microscope (Nikon, Tokyo, Japan).

### 2.6. Imaging Software

ImageJ software (version 1.52a) developed by National Institutes of Health and the Laboratory for Optical and Computational Instrumentation was used for adjusting and analyzing images.

### 2.7. Statistical Analysis

Student’s *t*-test and analysis of variance (ANOVA) in SPSS 25 (version 25.0; SPSS Inc., Chicago, IL, USA) were used for statistical analysis.

## 3. Results

### 3.1. The Changes of Serping1 and α-Syn in Colons

**Figure 1:**
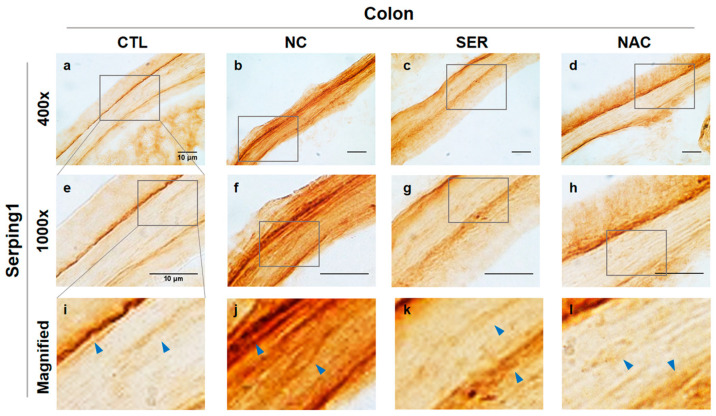
Immunohistochemical analysis of serping1 expressions in colon in each group. Serping1 observed in colonical tissues in each group (CTL; phosphate-buffered saline-treated group, NC; 1-methyl-4-phenyl-1,2,3,6-tetrahydropyridine (MPTP) 20 mg/kg and lipofectamine without short interfering RNA (siRNA)-treated group, SER; MPTP 20 mg/kg and lipofectamine with serping1 siRNA-treated group, NAC; MPTP 20 mg/kg and N-acetylcysteine (NAC)-treated group) are shown in (**a**–**l**) panels ((**a**–**d**), 400×; (**e**–**h**), 1000×). Stained serping1 is displayed by blue arrows in magnified panels in each group (**i**–**l**). Increased serping1 was observed in colonical smooth muscles of the NC group (**j**) panel compared to the serping1 in CTL, SER, and NAC groups (**i**,**k**,**l**) panels. Scale bar = 10 μm.

**Figure 2:**
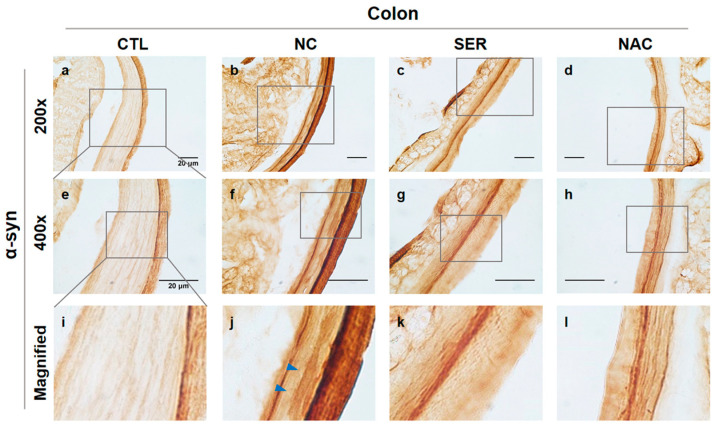
Immunohistochemical analysis of α-synuclein (α-syn) expression changes in colonical tissues of each group. α-syn observed in colonical tissues in each group (CTL; phosphate-buffered saline-treated group, NC; 1-methyl-4-phenyl-1,2,3,6-tetrahydropyridine (MPTP) 20 mg/kg and lipofectamine without short interfering RNA (siRNA)-treated group, SER; MPTP 20 mg/kg and lipofectamine with serping1 siRNA-treated group, NAC; MPTP 20 mg/kg and N-acetylcysteine (NAC)-treated group) are shown in (**a**–**l**) panels ((**a**–**d**), 200×; (**e**–**h**), 400×). Stained α-syn is displayed by blue arrows in magnified panels in each group (**i**–**l**). Increased α-syn was observed in colonical smooth muscles of the NC group (**j**) panel compared to the α-syn in CTL, SER, and NAC groups (**i**,**k**,**l**) panels. Scale bar = 20 μm.

Immunohistochemical analysis was performed to identify the changes of serping1 and α-syn expression patterns in each group (Figure 1 and Figure 2). The expression patterns were observed in colonical smooth muscles related to the intestinal peristalsis. The results observing Serping1 showed that increased serping1 expression was generated in the NC group compared to the CTL, SER, and NAC groups (Figure 1, indicated by blue arrows in i–l panels). This result indicated that serping1 siRNA injection decreased serping1 expression in the colonical smooth muscle in the SER group.

The results observing α-syn in colonical smooth muscles showed that increased α-syn expression levels were identified in NC group compared to the expressions in the CTL, SER, and NAC groups (Figure 2, indicated by blue arrows in the j panel). Founded on the reduced expression of serping1 in the SER group in Figure 1, this result indicated that α-syn was also decreased in colonical smooth muscles in which serping1 was reduced in the SER group.

**Figure 3:**
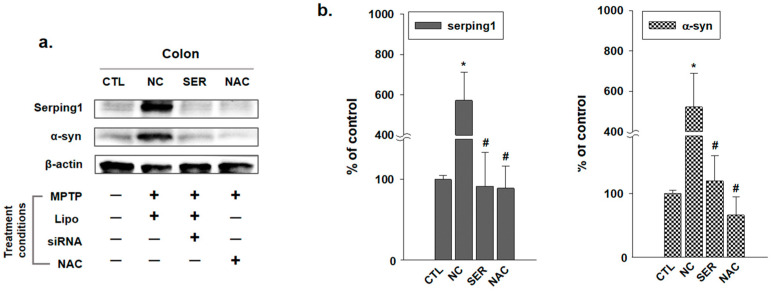
Serping1 and α-synuclein (α-syn) expression changes in intestine of each group. (**a**) Immunoblot analyses of Serping1 and α-syn in the colon of each group (CTL; phosphate-buffered saline-treated group, NC; 1-methyl-4-phenyl-1,2,3,6-tetrahydropyridine (MPTP) 20 mg/kg and lipofectamine (Lipo) without short interfering RNA (siRNA)-treated group, SER; MPTP 20 mg/kg and lipofectamine with serping1 siRNA-treated group, NAC; MPTP 20 mg/kg and N-acetylcysteine (NAC)-treated group) are shown, and the expressions of Serping1 and α-syn increased in NC group. However, the expressions of Serping1 and α-syn decreased in SER and NAC groups. (**b**) Immunoblot analyses of Serping1 and α-syn in the colon of each group were displayed by graphs. * *p* values compared to the value of CTL group. # *p* values compared to the value of NC group (*n* = 3, * *p* < 0.05, # *p* < 0.05).

Upon the immunohistochemical analyses observing serping1 and α-syn (Figure 1 and Figure 2), immunoblot analysis was also performed to examine the serping1 and α-syn expression levels in molecular change levels (Figure 3a). The results of the immunoblot analysis showed significantly increased serping1 and α-syn expression levels in the NC group. However, in the SER and NAC groups, serping1 and α-syn expression levels decreased significantly compared to the expression levels in the NC group (Figure 3b).

**Figure 4:**
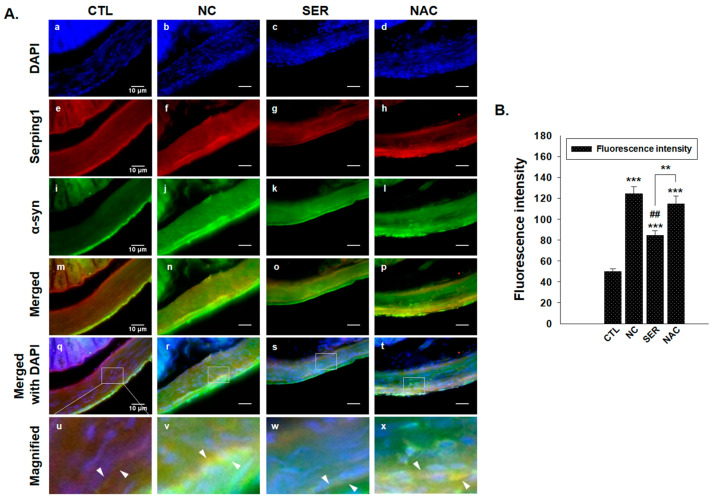
Immunofluorescence analysis of the changes of Serping1 and α-synuclein (α-syn) expressions in colon in each group. (**A**) Serping1 ((**e**–**h**); 400×) and α-syn ((**i**–**l**); 400×) were observed in colonical tissues in each group (CTL; phosphate-buffered saline-treated group, NC; 1-methyl-4-phenyl-1,2,3,6-tetrahydropyridine (MPTP) 20 mg/kg and lipofectamine without short interfering RNA (siRNA)-treated group, SER; MPTP 20 mg/kg and lipofectamine with serping1 siRNA-treated group, NAC; MPTP 20 mg/kg and N-acetylcysteine (NAC)-treated group). Nuclei stained by DAPI are also shown ((**a**–**d**); 400×) and merged panels observing serping1 and α-syn are displayed ((**m**–**p**); 400×) in each group. In addition, the panels merged with DAPI staining channel are also displayed ((**q**–**t**); 400×) and the (**q**–**t**) panels were magnified (**u**–**x**). The merged serping1 with α-syn were displayed by white arrows in NC and NAC groups in the magnified panels (**v**,**x**) panels. Overexpressed serping1 and α-syn were observed and significantly merged serping1 with α-syn appeared in NC group (**v**). Scale bar = 10 μm. (**B**) The quantification of merged fluorescence intensity showing the colocalization of serping1 and α-syn was indicated in a graph. *** *p* values compared to the value of CTL group. ## *p* values compared to the value of NC group. ** *p* value is comparing SER and NAC groups (*n* = 3, ** *p* < 0.005, *** *p* < 0.0005, ## *p* < 0.005).

Grounded on the changes of serping1 and α-syn levels, immunofluorescence analysis was conducted to observe both serping1 and α-syn simultaneously in colonical smooth muscle regions (Figure 4). In the NC group, significantly increased serping1 (Figure 4(f)) and α-syn (Figure 4(j)) appeared around nuclei stained by DAPI (Figure 4v; merged serping1 and α-syn that indicated by white arrows). However, serping1 and α-syn were not merged around nuclei in the CTL and SER groups (Figure 4(u,w)), as observed in the NC group (Figure 4B), and the NAC group did not show increased serping1 and α-syn though merged serping1, and α-syn appeared around the nuclei (Figure 4(x); merged serping1 and α-syn, indicated by white arrows). This result also revealed that serping1 and α-syn significantly increased in the NC group, which is consistent to the other analyses’ results.

According to the results, including immunohistochemistry, immunoblot, and immunofluorescence analyses, serping1 and α-syn consistently increased significantly in the NC group, and the expression patterns of serping1 and α-syn were similar, and also merged in the NC group of immunofluorescence analysis. Meanwhile, it is wondered how α-syn expressions change in the SN and ST regions in the brain.

### 3.2. The Changes of α-Syn in SN and ST Regions in the Brain

**Figure 5:**
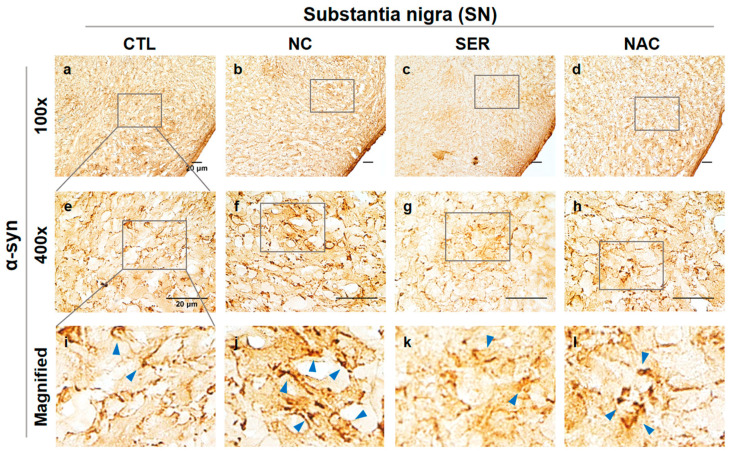
Immunohistochemical analysis of aggregated α-synuclein (α-syn) expression changes in substantia nigra (SN) of each group. α-syn observed in SN regions in each group (CTL; phosphate-buffered saline-treated group, NC; 1-methyl-4-phenyl-1,2,3,6-tetrahydropyridine (MPTP) 20 mg/kg and lipofectamine without short interfering RNA (siRNA)-treated group, SER; MPTP 20 mg/kg and lipofectamine with serping1 siRNA-treated group, NAC; MPTP 20 mg/kg and N-acetylcysteine (NAC)-treated group) are shown in (**a**–**l**) panels ((**a**–**d**), 100×; (**e**–**h**), 400×). Stained α-syn aggregations are displayed by blue arrows in magnified panels in each group (**i**–**l**) panels. Increased α-syn aggregations were observed in SN of the NC group (**j**) panel compared to the α-syn in CTL, SER, and NAC groups (**i**,**k**,**l**) panels. Scale bar = 20 μm.

**Figure 6:**
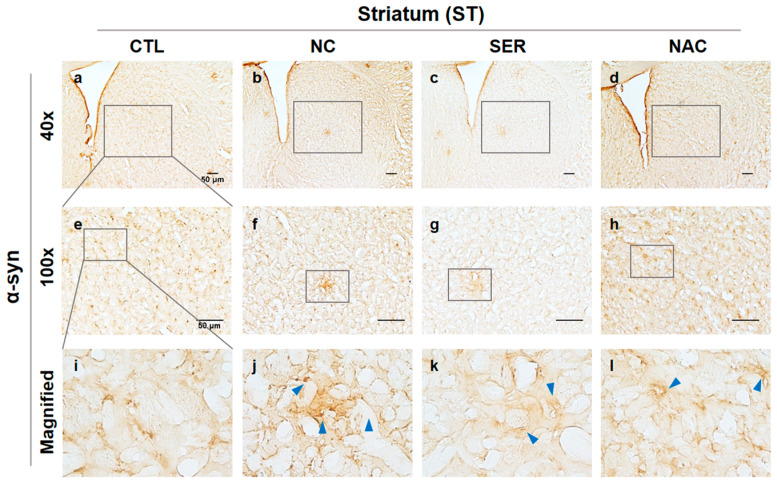
Immunohistochemical analysis of aggregated α-synuclein (α-syn) expression changes in striatum (ST). α-syn observed in ST regions in each group (CTL; phosphate-buffered saline-treated group, NC; 1-methyl-4-phenyl-1,2,3,6-tetrahydropyridine (MPTP) 20 mg/kg and lipofectamine without short interfering RNA (siRNA)-treated group, SER; MPTP 20 mg/kg and lipofectamine with serping1 siRNA-treated group, NAC; MPTP 20 mg/kg and N-acetylcysteine (NAC) treated group) are shown in (**a**–**l**) panels ((**a**–**d**), 40×; (**e**–**h**), 100×). Stained α-syn aggregations are displayed by blue arrows in magnified panels in each group (**i**–**l**) panels. Increased α-syn aggregations were observed in ST of the NC group (**j**) panel compared to the α-syn in CTL, SER, and NAC groups (**i**,**k**,**l**) panels. Scale bar = 50 μm.

To observe the changes of α-syn expressions in the brain, immunohistochemical analysis of α-syn expressions was performed in SN and ST regions in each group (Figure 5 and Figure 6). In the substantia nigra pars compacta (SNpc) region, the intensified aggregations of α-syn were observed in the NC group compared to other groups (Figure 5e–h). The aggregated α-syn oligomers are indicated by blue arrows in magnified images (Figure 5i–l). This result shows that aggregated α-syn increased in the NC group (Figure 5j). However, aggregated α-syn was alleviated in the SER group (Figure 5k) even more than the α-syn in the NAC group (Figure 5l).

In the ST region, the aggregated α-syn was also observed in the NC group compared to other groups (Figure 6e–h), and is indicated by blue arrows in magnified images (Figure 6i–l). This result also appeared to alleviate the aggregations of α-syn in SER group (Figure 6k), though it showed stronger aggregations of α-syn in the NC group like SNpc regions (Figure 6j). Founded on these results, it is wondered whether these change patterns would also be observed in molecular analysis.

**Figure 7:**
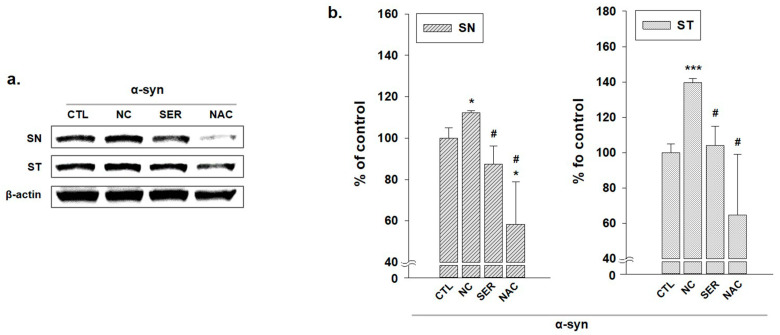
α-synuclein (α-syn) oligomer expression changes in striatum (ST) and substantia nigra (SN). (**a**) Immunoblot analyses of α-syn oligomers in ST and SN regions of each group (CTL; phosphate-buffered saline-treated group, NC; 1-methyl-4-phenyl-1,2,3,6-tetrahydropyridine (MPTP) 20 mg/kg and lipofectamine without short interfering RNA (siRNA)-treated group, SER; MPTP 20 mg/kg and lipofectamine with serping1 siRNA-treated group, NAC; MPTP 20 mg/kg and N-acetylcysteine (NAC)-treated group) are shown, and the generations of α-syn oligomer increased in NC group. However, the α-syn aggregation decreased in SER and NAC groups. (**b**) Immunoblot analyses of α-syn oligomers in ST and SN regions of each group are displayed by the bar graphs. * *p* values compared to the value of CTL group. # *p* values compared to the value of NC group (*n* = 3, * *p* < 0.05, *** *p* < 0.0005, # *p* < 0.05).

Immunoblotting analysis of α-syn expressions was performed in SN and ST regions in each group to confirm that the expression patterns of α-syn appeared, like in the immunohistochemical analyses results (Figure 7). In the blotting analysis, the expressions of α-syn oligomers increased in the NC group. However, in SER and NAC groups, α-syn oligomer expression levels were maintained as much as the expression levels of the CTL group, or decreased more than those of the CTL group in the SN and ST regions. According to these results, it can be deduced that the serping1 siRNA application also gives positive effects on the brain in an α-syn aggregation aspect.

Organizing these overall results, serping1 expressions increased in the NC group in the colon area, and α-syn expressions also increased in both the colon and brain areas in the NC group. However, applying serping1 siRNA decreased serping1 and α-syn expression levels in the colon, and furthermore, alleviated the aggregation of α-syn in the SN and ST regions of the brain in the SER group. The results of the SER group showed positive results comparable to the results of the NAC group.

## 4. Discussion

The study investigated the changes in serping1 and α-syn expression patterns in both the colon and brain regions of MPTP-induced PD mice. Immunohistochemical analyses were conducted to examine the changes of serping1 and α-syn expression patterns in each group in the colon (Figure 1 and Figure 2). Immunohistochemical analysis revealed increased serping1 expression in the colonic smooth muscles of the NC group compared to the CTL, SER, and NAC groups. Additionally, α-syn expression levels were higher in the NC group than in the CTL, SER, and NAC groups. And these results indicated that α-syn was also decreased in colonical smooth muscles where serping1 was reduced in the SER group. These findings are consistent with the results obtained from the immunoblotting assay, which showed increased serping1 and α-syn expression levels in the NC group and decreased expression levels in the SER and NAC groups (Figure 3). Furthermore, it was confirmed that there is a correlation between serping1 and α-syn in the NC group (Figure 4). It is worth noting that a previous report also explored the association between serping1 and α-syn [29].

These results suggest that serping1 siRNA knocked down the expression of serping1 in the colon, and this induced the decrease in α-syn expression in the SER group. Interestingly, the decreased expressions of serping1 were also observed in the NAC group. Known as an antioxidant, NAC gives a positive effect to inflammatory response [33,34], and decreases the angiotensin II receptor binding in smooth muscle cells [35]. This indicates that the actions of NAC are also related to the serping1 expressions, and serping1 could affect the inflammatory response and the extraction–relaxation in smooth muscles [24] in a reverse viewpoint. Therefore, it can be deduced that serping1 siRNA decreases α-syn in the colon as much as NAC. However, the results of the quantification analysis regarding the extent of serping1 and α-syn merging show a difference between the SER group and the NAC group (Figure 4B). It appears that NAC does not directly weaken the correlation between serping1 and α-syn. This indicates that the mechanism of action of NAC is not entirely identical to that of serping1 knockdown, although NAC can indeed influence the expression of serping1.

Meanwhile, α-syn aggregation was researched in the SN and ST regions in brains in each group. In the SN and ST regions, the increases in α-syn aggregation in the NC group and the decreases in α-syn aggregation in the SER and NAC groups were also observed (Figure 5 and Figure 6). Consistently, these results were also shown in the immunoblotting assay (Figure 7). In the immunohistochemical analysis, the α-syn aggregation was more alleviated in the SER group than even the NAC group (Figure 5 and Figure 6), though in the immunoblotting analysis result, α-syn aggregation decreased in the SER group as much as the CTL and NAC groups (Figure 7). These results suggest that the application of serping1 siRNA has a positive effect on reducing α-syn aggregation in the brain.

Grounded on the reports that synucleinopathy is related to gastrointestinal symptoms and α-syn as soluble oligomers might infiltrate the central nervous system [16,22], applying serping1 siRNA decreasing α-syn in colon could alleviate the α-syn aggregation in ST and SN regions. Although it has not yet been proved directly that soluble α-syn oligomers infiltrate the central nervous system, this study showed that the decreased α-syn by knocked-down serping1 in colon could affect α-syn aggregation in the brain.

In summary, the results consistently demonstrated increased serping1 and α-syn expression levels in the NC group in both the colon and brain regions. However, the application of serping1 siRNA led to decreased serping1 and α-syn expression levels in the colon and alleviated α-syn aggregation in the SNpc and ST regions of the brain in the SER group. These findings were comparable to the results observed in the NAC group.

The findings of this study provide valuable insights into the relationship between serping1 and α-syn in the context of PD. The observed decrease in serping1 expression following serping1 siRNA treatment suggests that serping1 plays a role in regulating α-syn expression. Moreover, the reduction in α-syn aggregation in the SER group indicates that targeting serping1 expression may have therapeutic potential for managing PD-related symptoms.

However, it is unclear how serping1 siRNA administration influences α-syn aggregation in the brain regions. Further investigations are needed to elucidate the molecular mechanisms underlying this phenomenon. Additionally, the study did not explore other factors related to PD, such as changes in tyrosine hydroxylase levels and inflammatory responses in dopaminergic neurons. Future studies should address these aspects to gain a comprehensive understanding of the role of serping1 in PD.

## 5. Conclusions

In conclusion, the expressions of serping1 and α-syn increased in the NC group but the expressions of serping1 and α-syn decreased in the SER and NAC group in colons in MPTP-induced PD mice. Applying serping1 siRNA knocked down the expression of serping1 in the colon, and this induced the decreased in α-syn expression in SER group. In addition, the increases in α-syn aggregation in the NC group and the decreases in α-syn aggregation in the SER and NAC groups were also observed in the brain. It seems that the application of serping1 siRNA could affect the decrease in α-syn aggregation in the brain. However, it is elusive how serping1 siRNA alleviates α-syn aggregation in the ST and SN regions of the brain. Factors related to PD, such as tyrosine hydroxylase changes and inflammatory responses in dopaminergic neurons, need to be more investigated in a following study.

## Data Availability

All necessary data are included in the paper.
